# Recent Advances in Molecular and Immunological Diagnostic Platform for Virus Detection: A Review

**DOI:** 10.3390/bios13040490

**Published:** 2023-04-19

**Authors:** Kieu The Loan Trinh, Hoang Dang Khoa Do, Nae Yoon Lee

**Affiliations:** 1 Department of BioNano Technology, Gachon University, 1342 Seongnam-daero, Sujeong-gu, Seongnam-si 13120, Gyeonggi-do, Republic of Korea; 2 NTT Hi-Tech Institute, Nguyen Tat Thanh University, Ward 13, District 04, Ho Chi Minh City 70000, Vietnam

**Keywords:** microfluidics, virus, immunoassay, molecular assay

## Abstract

Severe acute respiratory syndrome coronavirus 2 (SARS-CoV-2) has caused an ongoing coronavirus disease (COVID-19) outbreak and a rising demand for the development of accurate, timely, and cost-effective diagnostic tests for SARS-CoV-2 as well as other viral infections in general. Currently, traditional virus screening methods such as plate culturing and real-time PCR are considered the gold standard with accurate and sensitive results. However, these methods still require sophisticated equipment, trained personnel, and a long analysis time. Alternatively, with the integration of microfluidic and biosensor technologies, microfluidic-based biosensors offer the ability to perform sample preparation and simultaneous detection of many analyses in one platform. High sensitivity, accuracy, portability, low cost, high throughput, and real-time detection can be achieved using a single platform. This review presents recent advances in microfluidic-based biosensors from many works to demonstrate the advantages of merging the two technologies for sensing viruses. Different platforms for virus detection are classified into two main sections: immunoassays and molecular assays. Moreover, available commercial sensing tests are analyzed.

## 1. Introduction

Viruses are infectious microorganisms that contain genetic materials, either RNA or DNA, and are encapsulated by a protein coat called a capsid [1,2]. Viruses must replicate within host organisms and respond to the host metabolism. They can infect a wide range of life forms and cause cell changes or even cell death [3]. The early diagnosis of viruses is critical to save lives and control the spread of viral infection. Accurate, sensitive, and immediate virus diagnosis usually requires the laboratory testing of clinical samples to detect the presence of viruses [4]. Recently, there has been a major revolution in virus diagnostic methods that have played a leading role in controlling the spread of viral infections [5]. For virus detection, standard methods such as virus isolation [6], immune assays [7], nucleic acid amplification methods [8], and hemagglutination/inhibition assays [9] are usually employed. These methods typically require sophisticated and expensive instruments and trained operators to perform them. The assay for virus detection generally takes place in a central lab or hospital and requires a long analysis time, making it unsuitable for on-site detection in low-resource areas [10]. With the spread of severe acute respiratory syndrome coronavirus 2 (SARS-CoV-2), especially during the pandemic, rapid, sufficient, and sensitive diagnostic methods for virus detection are required to control the outbreak in both developing and developed countries.

Microfluidic technology manipulates fluids in channels with micrometer dimensions [11]. Inside a microfluidic chip, there are networks of microchannels engraved. The fluid is delivered into and out of the network of the channels through the inlet and outlet holes of the chip. Fluids are accurately manipulated to perform analysis by specific systems such as pumps, valves, and the micromixer system. Microfluidic chips have a high surface-to-volume ratio, which reduces the volume of reagents and increases the effectiveness of thermal and mass aspects. This technology has the potential to be applied in several fields, including physics, chemistry, biotechnology, and biochemistry [12]. The microfluidic system offers many advantages that provide high-throughput analysis and portability, decrease reaction time and sample and reagents used, enhance the control of fluid flow, and increase the mixing rate and sensitivity [13,14]. To design a microfluidic device, some dominant phenomena should be considered such as laminar flow, diffusion, surface tension, and electrokinetics. Many materials are good candidates for the fabrication of microfluidic devices. Among them, polymers are the most common because of their low price and well-established fabrication processes. Silicon-glass heterogeneous assembly is also a good choice for fabricating microfluidic chips which can resist chemicals and endure application of high pressure. Recently, paper has been considered an alternative choice in fabricating devices due to its biocompatibility, low cost, ease of use, and excellence in chemical analysis. A biosensor is an analytical device with biological recognition elements (such as nucleic acids, aptamers, antibodies, or enzymes). The recognition element is immobilized on a physicochemical transducer, and a detector is connected to identify the presence of specific analytes in the sample [15,16]. Owing to their portability, simplicity, accuracy, rapid analysis, and low cost, biosensors have wide applications in medical diagnosis, health care, food safety, and environmental monitoring [17,18,19]. Because of these advantages, integrating microfluidics and biosensors offers the benefit of merging biochemical and chemical processes into a single platform to produce a portable, disposable, accurate, specific, sensitive, high-throughput, and low-cost tool for viral infection monitoring. In this review, we comprehensively cover and update the research progress on the relevant microfluidic devices involved in virus detection in the past five years. The following section describes microfluidic platforms for several detection methods which can be broken down into (1) immunoassay-based biosensors for detecting antigens from virus particles and (2) molecular diagnostic biosensors for detecting DNA/RNA of viruses. Among immunoassay-based methods, the lateral flow assay (LFA) and Enzyme-Linked Immunosorbent Assay (ELISA), which are considered the gold standards for detecting protein components of viruses, are introduced together with their integration with biosensors. Antigen-antibody interaction-based virus sensing platforms other than LFA and ELISA are also reviewed to enhance some limitations of conventional LFA or ELISA. Another main component of the virus—nucleic acids—is discussed to target the molecules associated with virus detection in Section 2.2. The DNA/RNA-based detection methods are categorized as PCR and isothermal amplification reactions, and several compelling methods combining microfluidic technology for virus detection as well as the methods for detecting unamplified DNA/RNA are also introduced.

## 2. Virus Detection on Microfluidic Platform

### 2.1. Immunoassay Methods

Immunoassay methods are based on the highly specific interaction between the target antigen and corresponding antibody, which can rapidly recognize the virus protein and bind. Among the immunological detection methods developed to detect viruses, LFA and ELISA are most widely used because of their rapid and simple usage and high sensitivity. The following sections introduce many works of literature integrating biosensors with LFA and ELISA, as well as other immunological methods.

#### 2.1.1. Lateral Flow Assay-Based Tests

**Figure 1:**
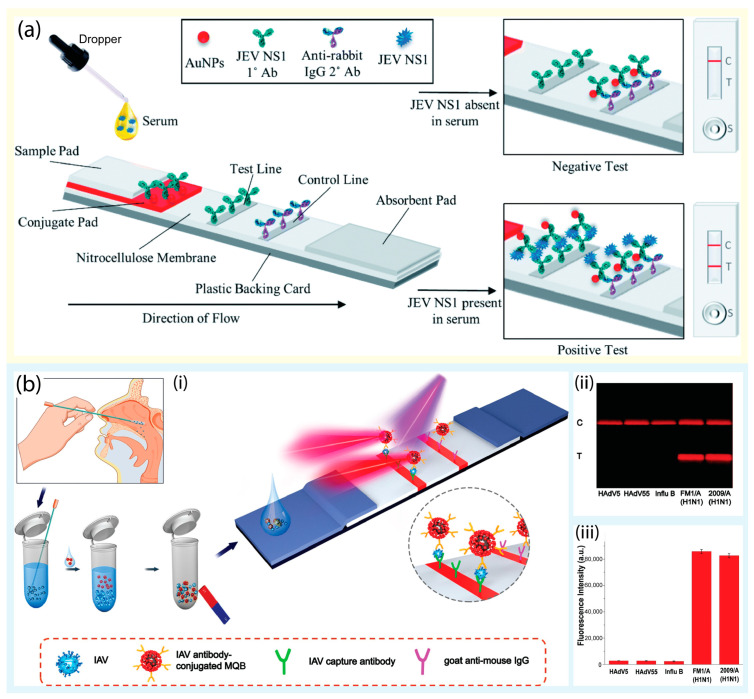
(**a**) Schematic representation of the colorimetric sandwich-based LFA for rapid detection of the Japanese Encephalitis virus using gold nanoparticles. Reprinted with permission from [25]. Copyright (2022) RSC publications. (**b**) Schematic representation of the MQBs-based fluorescent LFA for rapid and enrichment and ultrasensitive detection of influenza A virus in a human specimen. (i) Schematic diagram of influenza A virus detection using MQBs-based LFA. (ii) Images and (iii) corresponding fluorescence intensities of the LFA for H1N1 virions and is insensitive to other respiratory viruses. Reprinted with permission from [27]. Copyright (2020) Elsevier.

The lateral flow assay (LFA) is a widely accepted analytical approach that adopts paper-based biosensors to detect various analytes (e.g., heavy metals, proteins, and nucleic acids), owing to its advantages such as high specificity, sensitivity, user-friendliness, and affordability [20,21]. Accordingly, LFA-based devices hold great promise for point-of-care testing (POCT) applications in virus detection because of their low price and simple operation [22]. With the use of LFAs, various viruses (e.g., influenza A, B, and C; HIV; and Ebola) were successfully detected for on-site diagnosis without an expert. In addition, LFA has been the most used method for monitoring SARS-CoV-2, especially during the COVID pandemic [23]. In general, LFAs utilize antibodies as recognition elements and AuNPs as reporter probes, and the detection result is directly visualized on the test strip without requiring external energy sources [24]. For instance, Roberts et al. reported a portable LFA for on-site detection of the Japanese Encephalitis virus in serum (Figure 1a) [25]. Using AuNPs as immunoprobes, the test line turned red in the presence of the antigen, with a limit of detection (LOD) of 10 pg/mL within 10 min. In Le et al.’s study, the authors attempted to represent a dual-recognition element lateral flow assay for detecting influenza viruses [26]. In this study, the combination of an aptamer with an antibody was adopted to discriminate influenza virus types and subtypes, and a sensitivity of 90% was achieved as compared with quantitative real-time PCR (qRT-PCR). 

In another investigation, Wiriyachaiporn et al. used carbon nanotag-based LFAs for the rapid detection of influenza A virus in allantoic fluids [28]. In the presence of the virus, the formation of an antibody-antigen-carbon nanotag complex provided direct visualization of the virus through carbon nanotag accumulation on the test line. Thus, the detection limit of the virus reached approximately 350 TCID_50_/mL. Using carbon nanoparticles as reporters, this proposed LFA method could achieve better sensitivity and lower cost than other nanotag reporters (e.g., gold nanoparticles, and latex beads).

On the other hand, for quantitative detection, fluorescence-based LFA was also applied and demonstrated high sensitivity. A fluorescence-based approach was designed by Wang et al. in 2020 to detect SARS-CoV-2 RNA on an LFA strip [29]. The probe DNA-functionalized fluorescent nanoparticles were used to capture the RNA of SARS-CoV-2 as well as for signal amplification. In total, 734 clinical samples were evaluated, and the proposed LFA achieved a sensitivity of 100% and specificity of 99% compared to the results obtained from quantitative RT-PCR. Furthermore, Bai et al. constructed a magnetic quantum dot nanobead-based LFA (MQB-based LFA) for influenza A virus in clinical samples, with aLOD of 22 PFU/mL of H1N1 virahions within 35 min (Figure 1b) [27]. In addition, the high specificity of the MQB-based LFA for H1N1 virus strains against several other respiratory viruses was confirmed.

**Figure 2:**
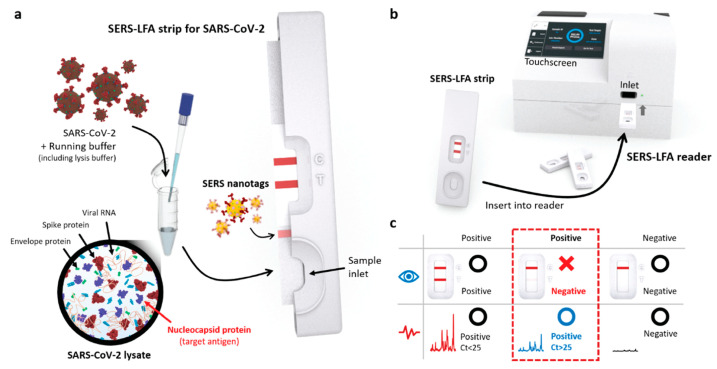
Schematic representation of the SERS-LFA system for the on-site detection of SARS-CoV-2. (**a**) The SERS-LFA strip. (**b**) The portable SERS-LFA reader. (**c**) The advantage of the portable Raman reader-based SERS-LFA system for on-site diagnosis compared to the commercial LFA strip. The red box indicates that most LFA assays showed false-negatives with low virus concentration (Ct > 25 in RT-PCR); however, the SER-LFAs showed positives, owing to their high sensitivity. Reprinted with permission from [37]. Copyright (2022) ACS publications.

On the other hand, the surface-enhanced Raman scattering (SERS) technique has been extensively adopted for virus detection, owing to its ultrahigh sensitivity and label-free detection. As a potent analytical technique for diagnostic investigations, many researchers have adopted SERS for the detection of various viruses such as SARS-CoV-2 [30], influenza viruses A and B [31], rotavirus [32], and hepatitis C virus [33]. Therefore, several studies have suggested that combining SERS with LFA can improve assay sensitivity. For example, Park et al. introduced a SERS-based LFA for the early diagnosis of influenza virus A [34]. In particular, instead of using gold nanoparticles, a SERS-active nano tag was embedded in the LFA strip, which led to an LOD as low as 1.9 × 10^4^ PFU/mL. Xiao et al. utilized an antibody-based SERS LFA to detect avian influenza virus H7N9 [35]. Core-shell AuAg^4−ATP^@AgNPs was first used as a Raman probe, and the LOD was as low as 0.0018 HAU with a total analysis time of 20 min. Lu et al. developed a dual-mode SERS-based LFA that can detect both SARS-CoV-2 and influenza A virus from clinical samples [36]. This approach can significantly reduce false-negatives compared with commercial colorimetric LFA assays. In addition, Young et al. developed a portable SERS-LFA system (SERS-LFA strip and reader) to resolve the sensitivity issues raised in commercial LFAs targeting SARS-CoV-2 from clinical samples (Figure 2) [37]. This system could detect the SARS-CoV-2 antigen on-site with high sensitivity within 15 min, with an LOD of approximately 3.53 PFU/mL.

In recent years, CRISPR-based LFAs have been developed to enhance the sensitivity and specificity of nucleic acid detection in biological samples containing a low number of targets [38,39]. In addition, a LFA strip combined with CRISPR/Cas9 allows multi-target detection and eliminates the need for instruments for nucleic acid-based detection [40]. In summary, LFA is a compelling approach for virus detection, especially multiplex detection. With the integration of other detection strategies, the sensitivity and specificity of LFAs can be enhanced, making commercialization more attainable.

#### 2.1.2. Enzyme-Linked Immunosorbent Assay-Based Tests

**Figure 3:**
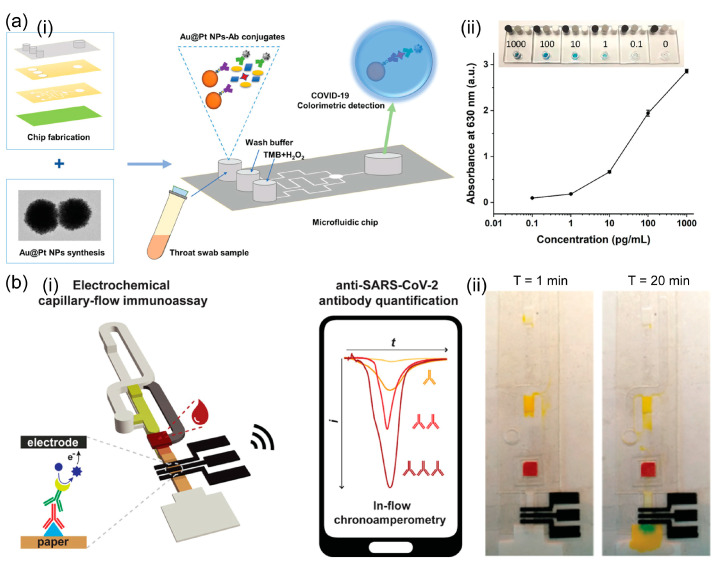
(**a**) Schematic representation of the platinum-decorated gold nanoparticle (Au@Pt NP)-based microfluidic chip immunoassay for COVID-19 detection. (i) Schematic diagram of COVID-19 immunoassay microdevice operation procedure. (ii) Analytical performance: Images of the microfluidic chip immunoassay and absorbance intensity obtained at different concentrations of N protein ranging from 0.1 to 1000 pg/mL. Reprinted with permission from [51]. Copyright (2022) ACS publications. (**b**) Schematic representation of the electrochemical capillary-flow immunoassay for SARS-CoV-2 detection. (i) Schematic overview of the entire smartphone-based detection system using electrochemical capillary-flow immunoassay. (ii) Sequential delivery of blood sample inside a microdevice within 20 min. Reprinted with permission from [57]. Copyright (2021) ACS publications.

The enzyme-linked immunosorbent assay (ELISA) is the gold standard for the detection of proteins, peptides, antibodies, and RNA/DNA of viruses because it has high sensitivity with a very low LOD and a high affinity for a surface epitope of viruses or bacteria [41,42,43]. However, commercial ELISA kits are relatively expensive and time-consuming (>120 min) [44,45]. To overcome these problems, many researchers have combined ELISA with microfluidics to improve the convenience of ELISA by making it a low-cost and rapid diagnostic tool [46,47,48]. Among other formats (such as direct, indirect, sandwich, and competitive ELISA), sandwich ELISA is the most widely adopted, owing to its highly efficient detection because two or more types of antibodies bind to samples [49,50]. For example, Wu et al. reported a sandwich ELISA-based microfluidic chip for nucleocapsid (N) protein detection in COVID-19 (Figure 3a) [51]. In this study, a synthesized platinum-decorated gold nanoparticle (Au@Pt NP) that displayed high catalytic activity was utilized for naked-eye detection less than 40 min, and the LOD was 0.1 pg/mL of N protein in a throat swab sample. Ma et al. reported an indirect multicolorimetric ELISA integrated with a microfluidic device for rapid visual detection of the hepatitis C virus, with an LOD of 9.1 ng/µL within 50 min [52]. Kasetsirikul et al. reported ELISA-based colorimetric detection of SARS-CoV-2 antibody, and the LOD was 9 ng/mL [53]. With the use of a paper-based microdevice, the sample volume needed for analysis was only 5 μL, and the results were validated by naked-eye readout within 30 min of analysis. Ozefe et al. also developed a micro-paper ELISA (µPISA) for rapid detection of the hepatitis C virus (HCV), and the LOD was approximately 10 ng/mL for HCV when a human blood plasma sample was used [54].

In another study, Song et al. introduced a digital protein microarray platform pre-equilibrium digital enzyme-linked immunosorbent assay (PEdELISA) microarray for the fast detection of four types of cytokines (IL-6, TNF-α, IL-1β, and IL-10) from COVID-19 patients for clinical applications, and an LOD as low as <0.4 pg/mL from clinical samples was achieved [55]. Significantly, this platform could achieve a rapid digital immunoassay with a clinically relevant fM–nM dynamic range without losing signal-sensing linearity by combining single-molecular counting with early pre-equilibrium reaction quenching. Furthermore, Clark et al. presented an electrochemical capillary-driven immunoassay device for directly detecting SARS-CoV-2, influenza A, and sindbis virus [56]. In particular, the automated sandwich ELISA sequentially introduced all reagents using a flow device, enhancing user-friendliness and reducing the analysis time and cost of this platform. In another investigation, Samper et al. reported a novel low-cost electrochemical capillary-flow device for the rapid quantification of IgG antibodies targeting SARS-CoV-2 protein in human whole blood samples based on a competitive ELISA method, and the LOD was approximately 5 ng/mL (Figure 3b) [57]. Recently, Samper et al. also used electrochemical immunoassays for the rapid detection of SARS-CoV-2 in nasopharyngeal samples within 70 min [58]. This device could achieve high specificity using a sandwich ELISA for capturing the antigen of the virus, and the LOD was approximately 50 PFU/mL from 20 µL of the virus sample.

Wang et al. developed an ELISA-based microfluidic immunoassay for the rapid, high-throughput, and ultrasensitive detection of multiple SARS-CoV antigens and antibodies in serum samples [59]. By integrating graphene oxide quantum dots and microdevices, this biochip could simultaneously detect 60 serum samples in a single assay. It only takes 10 min for qualitative detection with high sensitivity (LOD ~0.3 pg/mL). 

To improve the efficiency of virus diagnosis, Gong et al. proposed a paper-based microfluidic ELISA for SARS-CoV-2 detection in human blood [60]. In this study, the microfluidic platform was used to collect the serum by a pulling-force spinning top, and then the IgA/IgM/IgG was measured by ELISA without the use of any instrument. This device achieved a high specificity and sensitivity (>>99% accuracy) for confirming SARS-CoV-2 infection. In addition, the fabrication cost of this device was less than 5.00 USD, which could provide an excellent option for the detection of other viruses in low resource-setting areas or even home diagnosis of SARS-CoV-2 or other viruses. In another study, Chen et al. fabricated an electricity and external instrumentation-free 3D origami paper-based biosensor-based ELISA for the detection of HIV, and the limit of detection was 0.03 ng/mL [61]. Thus, ELISA-based microfluidic immunoassay opens a new door for commercializing a portable, cost-effective, and rapid immunosensing platform with ultra-sensitivity, on-site detection, and use in low-resource setting areas.

#### 2.1.3. Others

Other than the abovementioned methods, alternative approaches have been exploited to achieve a higher degree of sensitivity in determining the presence of virus antigens. These include electrochemistry-based methods (e.g., field-effect transistor (FET)), optical-based methods (e.g., SERS), and more recently, terahertz-wave-based methods. The following section discusses about the adaptation of these techniques in immuno-based sensors.

**Figure 4:**
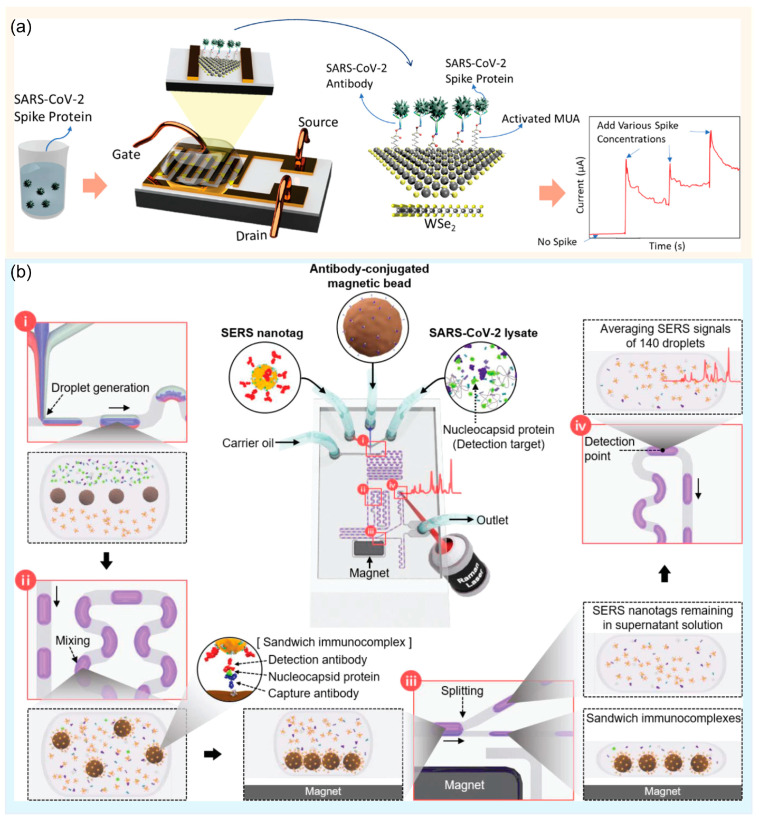
(**a**) Schematic representation of the FET-based biosensor for the rapid and sensitive detection of SARS-CoV-2 in vitro. Reprinted with permission from [68]. Copyright (2022) ACS publications. (**b**) Schematic representation of the microdroplet SERS sensor for the immunodiagnostic test of SARS-CoV-2. (i) Droplet generation. (ii) Droplet mixing. (iii) Droplet splitting. (iv) Optical signal measurement. Reprinted with permission from [69]. Copyright (2022) Elsevier.

Field-effect transistor (FET) biosensors have been actively applied for virus detection, especially during the COVID-19 pandemic, owing to their small size, label-free nature, multiplex detection, and ultra-high sensitivity [62,63,64]. Seo et al. developed a graphene-based FET biosensing device for the early diagnosis of SARS-CoV-2 in clinical samples [65]. This FET sensor achieved a high sensitivity (1 fg/mL) by integrating the SARS-CoV-2 spike antibody with graphene. Chen et al. reported that reduced graphene oxide-based FET were useful as conducting channels in FET-based biosensors for real-time detection of the Ebola virus in clinical samples, and the limit of detection was approximately 1 ng/mL with a real-time response [66]. Wasfi et al. also used a graphite oxide-based FET for real-time detection of the COVID-19 spike protein antigen, and the limit of detection was approximately 1 fg/mL of the COVID-19 spike antigen in phosphate-buffered saline [67]. In another study, Fathi-Hafshejani et al. reported a novel WSe_2_-based FET biosensor for the rapid detection of SARS-CoV-2 in vitro (Figure 4a) [68]. This FET could achieve such a high sensitivity (LOD = 25 fg/mL) by functionalizing WSe_2_ monolayers with a monoclonal antibody against the SARS-CoV-2 spike protein. Therefore, this platform shows immense potential for immunological diagnosis and can be applied for rapid virus detection in clinical diagnosis, on-site detection, and POCT.

Optical-based methods were also exploited to detect viruses. Park et al. introduced a SERS microdroplet sensor for the rapid detection of SARS-CoV-2 using magnetic beads (Figure 4b) [69]. In this study, using a combination of SERS and microdroplets, this platform dramatically improved the limit of detection (0.22 PFU/mL) within 10 min of the total assay time. This SERS-based microdroplet sensor has high potential for developing a new POCT for the detection of viruses, including SARS-CoV-2. Furthermore, Achadu et al. reported a SERS-based biosensor for ultrasensitive immunoassays of hepatitis E virus or norovirus using molybdenum trioxide quantum dots (MoO_3_-QDs) as SERS nanotags. In addition, this SERS-based biosensor achieved a signal enhancement factor, and the limit of detection was approximately 6.5 and 8.2 fg/mL for the hepatitis E virus and norovirus in the fecal specimens and clinical samples, respectively.

More recently, there have been efforts to develop more direct and non-destructive methods for identifying viruses, and terahertz-based spectroscopy has gained attention [70,71,72]. Terahertz waves have a low photon energy of around 4 meV for 1 terahertz, which avoids damaging analytes from heat and ionization. In addition, the terahertz range includes binding energies between major biological molecules such as hydrogen bonding, which can be engaged in the investigation and identification of unique spectral properties of biological matters including viruses. However, the strong absorption to water molecules limits their application to hydrated samples, and low sensitivity and specificity restrict the virus identification, as demonstrated by Lee et al., where the H9N2 virus pellet showed no spectral difference from its control [73]. They further improved its detection sensitivity by constructing a terahertz metamaterial-based sensor, which can greatly enhance the electric field depending on the incoming radiation of terahertz wavelengths. The metamaterial chip was designed to have three different resonance frequencies which aided in the detection of optically unknown viruses. Ahmadivand used a toroidal metamaterial sensor with bimetallic asymmetrical planar resonators to detect the envelop protein of the Zika virus [74]. Zika virus antibody was immobilized on the surface of the structure, and the shift in toroidal resonant frequency in response to Zika virus envelop protein added to the chip was measured to obtain ~24 pg/mL LOD. Results from other studies suggest that incorporating gold nanoparticles (AuNPs) into terahertz sensors can remarkably enhance their sensitivity [75]. Shi constructed all-dielectric terahertz metamaterial sensors to detect human influenza hemagglutinin tag protein, assisted by AuNPs functionalized with the specific antibody [76]. According to the results, 2.66 times higher sensitivity was achieved when compared to the counterpart without the functionalized AuNPs, and its specificity was validated by comparing the target against bovine serum protein, ovalbumin, and whey proteins.

**Figure 5:**
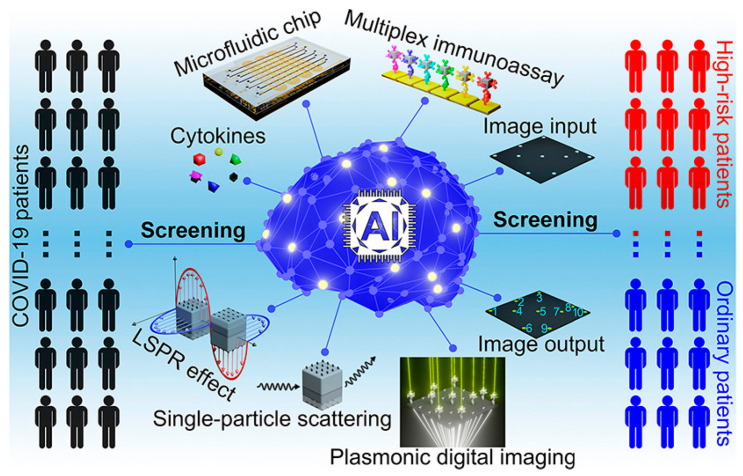
Schematic representation of the machine-learning-assisted microfluidic nanoplasmonic digital immunoassay for high-throughput, multiplex cytokine detection in COVID-19 patients. Reprinted with permission from [78]. Copyright (2021) ACS publications.

In addition, “machine learning” has been intensively used to help enhance the performance of microfluidics with higher sensitivity and specificity [77]. Gao reported a machine-learning-assisted microfluidic nanoplasmonic digital immunoassay for cytokine monitoring in COVID-19 patients (Figure 5) [78]. This study used a facile one-step sandwich immunoassay format for the simultaneous detection of six cytokines (IL-1β, IL-2, IL-6, IL-10, TNF-α, and IFN-γ) in a single run using only 3 μL serum samples, and the LOD was approximately 0.46–1.36 pg/mL. The combination of microfluidic technology and machine learning effectively achieved the high-throughput detection of multiple immune biomarkers in a rapid, sensitive, selective, accurate, and easy-to-implement manner to be exploited as an advanced point-of-care (POC) detection platform for immune biomarkers effectively applicable for the early-stage diagnosis of diseases. In another study, Teengam et al. introduced a smartphone-based portable immunosensing device for the detection of HBV, and the limit of detection was about 0.17 µg/mL of HBsAg in the clinical samples [79]. With the use of smartphones, this low-cost electrochemical immunosensor not only provides real-time results with high sensitivity and portability, but could also help to upload and share the results information for supporting treatment or outbreak prevention. 

### 2.2. Molecular Methods

Early diagnosis can effectively control the spread of viral infection. Therefore, reliable and rapid screening methods are needed. Immunoassays and molecular methods are the most common technologies used for virus detection [80]. Although immunoassays are simple and rapid, they still require more sensitivity and accuracy in the initial stages of infection. Molecular methods that detect viruses based on their nucleic acids are highly sensitive and specific [81,82]. Microfluidic-based biosensors for molecular methods have been developed based on the standard approach of nucleic acid analysis involving conventional PCR and isothermal amplification. These methods were adapted from macroscale molecular methods, mainly by integrating multiple steps into a microfluidic platform. The downscaling process benefits sample and reagent savings, decreases contamination, and has a rapid turnaround time. In this section, we summarize several examples of microfluidic devices for virus detection based on molecular methods.

#### 2.2.1. PCR

**Figure 6:**
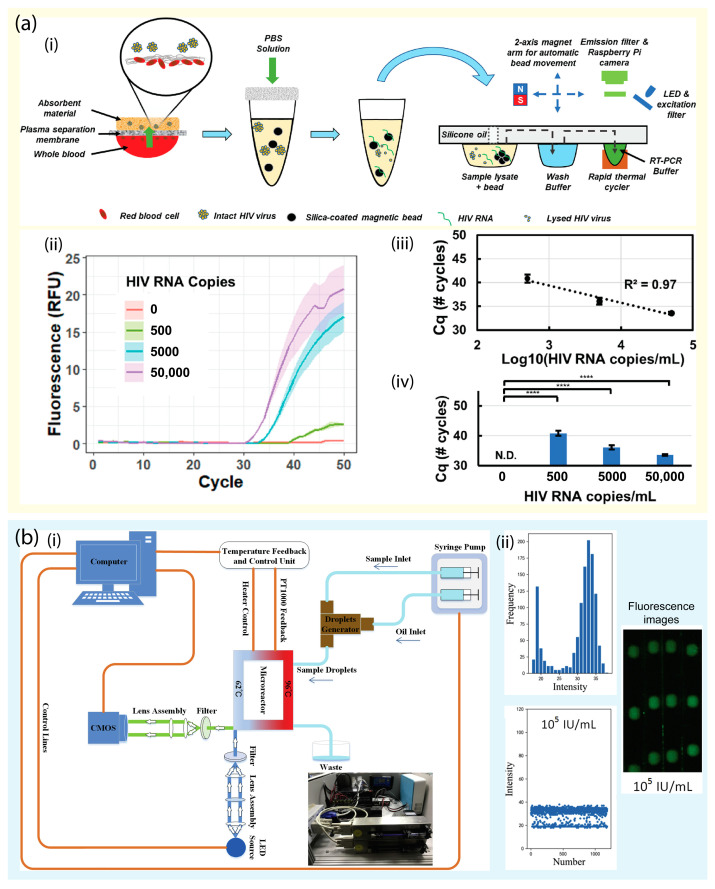
(**a**) Schematic representation of the POC HIV virus. (i) Working principle of the microdevice operation for HIV detection. (ii) Amplification curves. (iii) Cq values. (iv) LOD evaluation at 500 HIV RNA copies/mL. N.D. stands for non-detected. **** indicates *p* < 0.0001. Reprinted with permission from [88]. Copyright (2022) ACS publications. (**b**) A fully integrated continuous-flow digital PCR device for hepatitis B detection. (i) Schematic representation of the system of continuous-flow digital PCR instrument. (ii) The digital PCR detection results: the distribution, frequency, and real image of the fluorescence intensity of the droplets. Reprinted with permission from [90]. Copyright (2020) Elsevier.

The gold standard for detecting viral infection is a nucleic acid amplification test using PCR [83,84]. These methods offer excellent accuracy, sensitivity, specificity, and time efficiency. However, the major disadvantage of PCR is the requirement of sophisticated equipment for the reaction [85,86]. Owing to the success of conventional PCR, many microfluidic platforms have been integrated with PCR for virus detection without the need for a complicated thermocycler. Research published in 2022 introduced a portable and magnetofluidic cartridge platform for the multiplexed detection of SARS-CoV-2 [87]. The platform can identify SARS-CoV-2 variants and screen for influenza A and B. Automated PCR was integrated for virus detection within 30 min [87]. Clinical samples, including saliva and nasopharyngeal samples, were collected for testing. The magnetofluidic cartridge ensured transportation of the sample for nucleic acid purification and amplification. In another study, a platform for sensitive POC HIV load quantification was developed (Figure 6a) [88]. HIV can be quantified from the blood. The device has a plasma separation membrane and absorbent material. After plasma separation, the viral RNA was extracted using a magnetofluidic cartridge. Purified RNA was then amplified and quantified using a portable instrument for RT-PCR within 15 min, and the limit of detection was approximately 500 HIV RNA copies/mL. A ”FilmArray” was developed to detect and identify 21 common viral and bacterial respiratory pathogens from one sample in one hour. The platform combined nucleic acid extraction and PCR to simultaneously screen a wide range of nucleic acid targets [89]. A fully automatic integrated digital PCR system has been proposed for the quantitative detection of hepatitis B. The greatest advantage of this system is that it is an automatic and fully integrated all-in-one system. Unlike existing digital PCR systems, which require many separate devices, including sample loading, droplet generation, PCR reaction, and signal analysis, this system simplifies multi-step operation on one platform (Figure 6b). For analysis, the sample was added to the entrance of the instrument, and the test results were directly obtained [90].

#### 2.2.2. Isothermal Amplification

**Figure 7:**
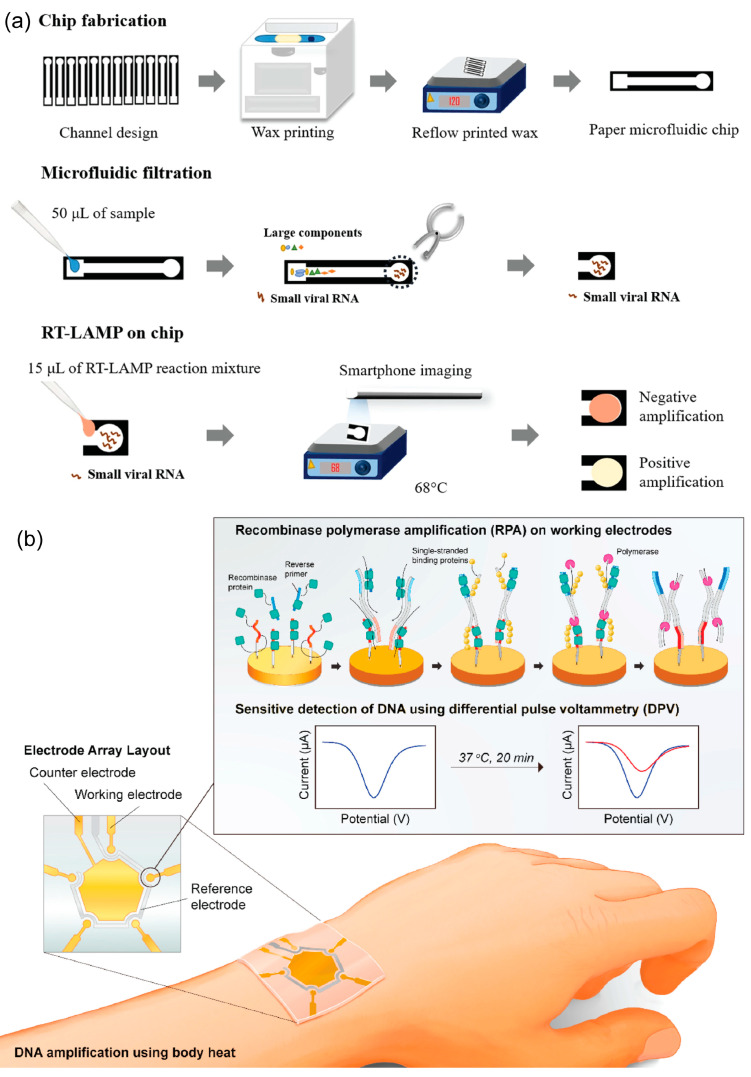
(**a**) Schematic representation of the paper microfluidic RT-LAMP assay for rapid detection of the Zika virus. Reprinted with permission from [106]. Copyright (2018) Springer Nature. (**b**) Schematic representation of the electrochemical biosensor combined with isothermal amplification for COVID-19 detection. Reprinted with permission from [102]. Copyright (2021) Elsevier.

Isothermal nucleic acid amplification is an alternative method to conventional PCR. Owing to the use of a thermocycler, PCR has limitations in its application for POCT. Meanwhile, isothermal amplification requires only one constant temperature for the reaction; therefore, it eliminates the need for complicated temperature control [91,92,93]. Many isothermal amplification methods have been developed and extensively applied to POCT, including nucleic acid sequence-based amplification (NASBA), recombinase polymerase amplification (RPA), loop-mediated isothermal amplification (LAMP), helicase-dependent amplification (HDA), and recombinase aided amplification (RAA) (Figure 7) [94,95]. Currently, isothermal amplification has attracted significant attention for its wide application in the diagnostic field, especially for SARS-CoV-2 detection, without the need for sophisticated instruments [96]. LAMP is a promising candidate for developing rapid and simple molecular-based microfluidic-based biosensors to detect viral infections [97]. This technique employs between two and three primer sets to recognize many regions of the target sequence with high specificity and sensitivity. The temperature required for the reaction was approximately 60–65 °C with a reaction time of less than 1 h. Owing to these advantages, LAMP has been employed to develop microfluidic-based biosensors. A POCT platform was developed to detect SARS-CoV-2 with a high accuracy, specificity, and sensitivity. The LAMP assay was used to detect SARS-CoV-2 [98]. The platform, in which sample transportation, extraction, and amplification steps were integrated into a disposable cartridge, was ultra-portable. The entire process for sample analysis was controlled by a unit with a pocket size and was operated by a battery. Colorimetric results were obtained within 35 min [98]. A paper-based device has been introduced to detect SARS-CoV-2 in human saliva. RT-LAMP was integrated into the device to target SARS-CoV-2. The colorimetric signal obtained from the reaction enabled a user-friendly response. Within 60 min, the device could detect the virus in the sample without sample preparation, with a high specificity of 100% and high sensitivity of 97%. The LOD for this device was 200 copies/μL [99]. In another study, Wang introduced a dual microchip (Dµchip) and portable detection device for simultaneous detection of SARS-CoV-2, influenza viruses A, H1N1, H3N2, and influenza viruses B using RT-LAMP and Cas12a assays, and the LOD was 10 copies/sample [100]. 

RPA employs a recombinase and polymerase for amplification. Primer hybridization is catalyzed by recombinase. The recombinase-primer complex scans through double-stranded DNA and searches for homologous sequences. Upon recognition of the cognate site, the complex promotes strand exchange. Single-stranded DNA-binding proteins help stabilize the resulting DNA structure. DNA polymerase catalyzes DNA prolongation. The reaction temperature was quite low—approximately 37–42 °C—and results were obtained in less than 30 min [101]. An electrochemical biosensor was developed to detect SARS-CoV-2 rapidly and accurately. The sensor was combined with RPA on a multi-microelectrode platform and targeted multiple SARS-CoV-2 genes. Primer sets for each gene were immobilized on the electrodes, and the production of amplicons resulted in a significant reduction in the current density. Signals from different electrodes were recorded using differential pulse voltammetry. *RdRP* gene and *N* gene of the virus could be detected at a low level with the limit of detection of 0.972 fg/μL and 3.925 fg/μL, respectively [102]. A wearable device was used to detect the HIV-1 DNA. The microfluidic device was combined with the RPA, and body heat was used as the heat source for amplification. A cellphone-based fluorescence detection system was integrated into the device to realize a portable, electricity-free device for the POCT of infectious pathogens in low-resource areas. The LOD of the device was approximately 10^2^ copies/mL within 24 min [103]. 

Rolling circle amplification (RCA) used unique DNA or RNA polymerases and circular DNA templates to amplify short DNA or RNA. The RCA product had a concatemer structure. The structure contained tandem repeats with sequences complementary to the circular template [104]. A microfluidic system using mesh-based RCA was developed to detect SARS-CoV-2. The most exciting feature of this approach is that the system combined RCA, DNA gelation, blockage by gelation in the mesh micropores, and hydrostatic microfluidics. The system offered a rapid and ultra-sensitive detection of SARS-CoV-2 with a DNA concentration of 3–30 aM in 5 to 15 min [105]. 

#### 2.2.3. CRISPR-Assisted Method

**Figure 8:**
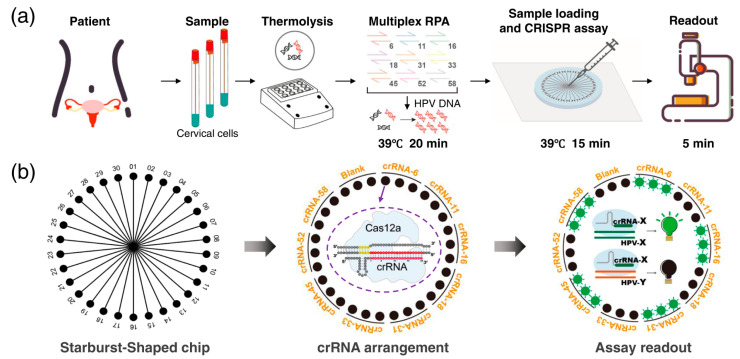
Schematic representation of the microfluidic device combined with CRISPR-Cas12a and multiplex recombinase polymerase amplification for detection of human papillomavirus (HPC). (**a**) Brief overview of the steps involved in the process. (**b**) On-chip testing principles. Reprinted with permission from [116]. Copyright (2022) Springer Nature.

Clustered regularly interspaced short palindromic repeats (CRISPR) and CRISPR-associated (Cas) protein systems were first discovered in 1987 [107]. The CRISPR system, originating from archaea and bacteria, is the immune mechanism of these microorganisms. The system contains the CRISPR gene and CRISPR-Cas proteins, which can cleave specific nucleic acids under the guidance of RNA [108]. Recently, CRISPR-Cas systems have been widely applied in the diagnostic field because of their specificity, ease of use, and programmability [109,110,111]. In this section, we discuss the different Cas enzymes and their applications in diagnostic assays. Based on genes encoding effector modules, the CRISPR-Cas system can be divided into two main classes: “class 1” and “class 2” [112]. Furthermore, based on the organization of their loci and proteins, these classes can also be subdivided into distinct types. The class 1 system, which employs a complex of multiple effector proteins, includes types I, III, and IV. The class 2 system encompasses single crRNA-binding proteins, such as Cas9, Cas12, and Cas13. Because of the activities of different Cas systems, class 2 systems are widely used for diagnostic purposes. For example, CRISPR/Cas13 can be used to target single-stranded RNA, and CRISPR/Cas14 can be used to target single-stranded DNA [113]. In addition, the use of the CRISPR/Cas system for nucleic acid detection can achieve multiplexing and high sensitivity down to the attomolar level [113]. Therefore, the integration of CRISPR/Cas into microfluidic-based biosensors may provide accurate, highly sensitive, rapid, and powerful tools for nucleic acid detection (Figure 8) [114,115,116]. A face mask with a SARS-CoV-2 sensor was developed for wearable, simple, and noninvasive detection. The detection process required minimal user intervention with only one button-pressing step [117]. The sensor combined RT, RPA, and CRISPR/Cas12a for SARS-CoV-2 detection. The limit of detection of the sensor was 500 viral copies. An automated system was developed to detect Ebola RNA with the aid of RNA-guided RNA endonuclease Cas13a. The microfluidic system provided an automatic mixing and hybridization strategy for sample analysis. The generated signals were measured using a custom-integrated fluorometer [118]. Another study introduced a rapid assay for the sensitive detection of SARS-CoV-2 by integrating isothermal amplification with CRISPR-Cas12. The target RNA was amplified by reverse transcriptase recombinase polymerase and recognized by the CRISPR assay. The signal was collected by fluorescence measurements or a lateral flow strip [119]. Recently, Zhou et al. reported a heating-membrane-assisted multiplexed microfluidics for the fast and low-cost detection of HPV subtypes using a combination of RPA and CRISPR assays [120]. This device could simultaneously detect HPV16 and HPV18 within 30 min, and the LOD was approximately 1 × 10^–18^ M using plasmids. In addition, Zhao et al. developed a dual-droplet device for simultaneous detection of HPV16 and HPV18 by combining the CRISPR-Cas12a system with a multiplexed RPA assay, and the LOD was about 1 copy/reaction [121]. Two types of influenza virus, A and B, were successfully detected using a rapid on-site assay. This assay was developed based on the activity of the CRISPR/Cas system in combination with RT-LAMP. The lateral test strips provided a naked-eye read-out. The results were obtained in less than 85 min with high sensitivity and specificity [122]. An autonomous lab-on-paper platform has been developed to detect SARS-CoV-2 rapidly. The assay combined RT-RPA and CRISPR-Cas12a to simultaneously identify multiple genes, including the nucleoprotein gene, spike gene of the virus, and human housekeeping RNAse P gene. The assay can detect down to 10^2^ copies of viral RNA in less than one hour [123]. A diagnostic platform called microfluidic Combinatorial Arrayed Reactions for Multiplexed Evaluation of Nucleic acids (mCARMEN) was proposed to effectively detect viruses and their variants. The platform combined microfluidic technology with the CRISPR/Cas system to test up to 21 viruses. Many samples collected from clinical settings were tested to prove the effectiveness of the system [124]. A microfluidic system (MAPnavi) was introduced to accurately and sensitively detect multiple respiratory viruses in less than 40 min. The system integrated nested RPA with CRISPR/Cas12a to achieve a low detection limit of 50–200 copies/mL. Swab samples collected from COVID-19 patients were used to demonstrate the success of this approach [125]. 

### 2.3. Others

In addition to nucleic acid amplification and immune assay methods, other approaches, including electrochemical detection, advanced microscopy, and plasmonic sensing, have been applied to detect various viruses.

An inexpensive and rapid assay for the colorimetric detection of SARS-CoV-2, termed MARVE (for multiplexed, nucleic-acid-amplification-free, single-nucleotide-resolved viral evolution), was developed to detect SARS-CoV-2 variants at the single-nucleotide level. For user-friendly and cost-effective purposes, the assay was integrated into a foldable paper platform. The assay detected viral variants based on a nucleic acid amplification-free strategy. Upon recognizing the specific sequence of the virus, an enzyme-based assay generated a colorimetric signal to realize a simple method for the result readout [126].

**Figure 9:**
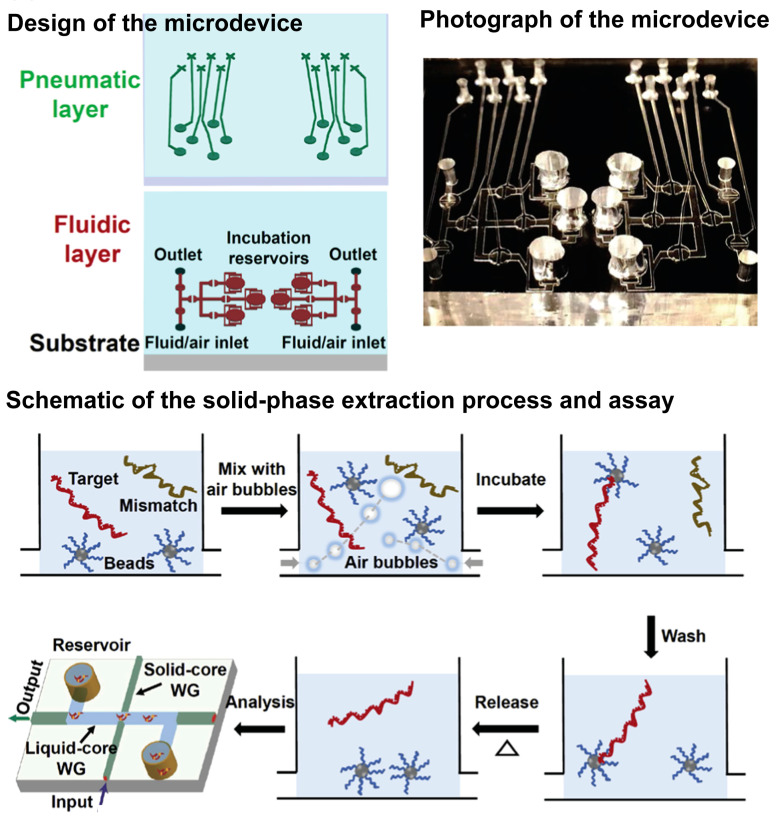
Schematic representation of the fabrication method for the automated microfluidic sample preparation multiplexer with lifting-gate microvalves for Ebola virus detection. Reprinted with permission from [129]. Copyright (2017) Elsevier.

A sample preparation multiplexer (SPM) was introduced to improve the speed, extraction efficiency, and throughput of the optofluidic system. The system increased the efficiency and speed of the target capture by employing metered air bubbles. The bubbles stirred up the magnetic beads in which the capture probes were immobilized (Figure 9) [127]. An impedance biosensor was developed to detect the H5N1 avian influenza virus. The microfluidic flow cells and microelectrodes were integrated into the biosensor. The DNA aptamer was immobilized on the electrode to identify specific targets. When the virus was specifically captured on the microelectrode surface, the magnitude of impedance increased. This biosensor can detect viruses within 30 min [128]. An electromechanical biosensor was used to detect SARS-CoV-2 RNA. The biosensor was incorporated into a portable device. The detection was performed using a molecular system. The system consisted of an aptamer probe bound to a cantilever made of flexible single-stranded DNA. The cantilever was linked to a tetrahedral double-stranded DNA structure. The system offered an ultrasensitive diagnostic tool with 1–2 copies in 100 μL in less than 4 min [129].

## 3. Microfluidic Assists in the Pandemic Era

In late 2019, the COVID-19 outbreak caused by SARS-CoV-2 spread rapidly across the globe, leading to a great crisis in global health, life, and the economy. To limit the damage cause by COVID-19 and other infectious diseases, effective and rapid diagnostic tools are necessary. During the pandemic, the market witnessed a substantial number of commercial assays for the rapid detection of the virus. Technological innovations have played an important role in the early detection of viruses. The integration of microfluidic-based biosensors offers many advantages for infection monitoring compared with conventional laboratory-based methods, especially for applications in low-resource areas. Its advantages include ease of use, low cost, high throughput, rapid analysis time, affordable accuracy and sensitivity, and portability. To guide the development of diagnostic tools in low resource areas, The World Health Organization has set guidelines referred to as “ASSURED”—(a) affordable, (b) sensitive, (c) specific, (d) user-friendly, (e) rapid and robust, (f) equipment-free, and (g) deliverable to end users [130,131].

Over the past two decades, the growth rate of microfluidic products in the medical diagnosis market has been sharply increasing, which indicates the potential for the commercialization of microfluidics. Driven by the great demand for portable, wearable devices, point-of-care testing, and personal healthcare, the global microfluidic components market is expected to grow from 20.7 billion USD in 2021 to 23.2 billion USD in 2026 [132]. Several ventures have commercialized a variety of microfluidic platforms to monitor different types of targets, including pathogens and cancer markers (Table 1).

In this section, we summarize some outstanding products for virus detection [133,134]. We mainly focused on common microfluidic platforms for virus detection, especially during the pandemic period. 

Elecsys^®^ Anti-SARS-CoV-2, developed by Roche, is an immunoassay used for the qualitative detection of SARS-CoV-2 antibodies. Human serum or plasma samples were used for testing. The test was intended to indicate whether the user has had a recent or prior infection. The analysis time for the test was approximately 18 min [135].

The Cobas^®^ SARS-CoV-2 test is a qualitative assay developed by Roche that targets SARS-CoV-2 based on nucleic acid amplification reactions. A fully automated cobas^®^ 6800/8800 system was used for the test. The samples collected for the test were oropharyngeal, nasal, and nasopharyngeal swab [136].

The ID NOW™ COVID-19, produced by Abbott, is one of the leading molecular point-of-care platforms for COVID-19 diagnosis. The assay was performed using the ID NOW Instrument. The nucleic acid isothermal amplification technology integrated into the assay targeted a unique region of the RdRp segment of the virus. The results were obtained within 13 min [137].

Xpert^®^ Xpress CoV-2 *plus* is a rapid, real-time RT-PCR test developed by Cepheid. This was intended to qualitatively detect nucleic acid of SARS-CoV-2. The samples used for the test were nasopharyngeal swabs, anterior nasal swabs, mid-turbinate nasal swabs, oropharyngeal swabs, and nasal wash/aspirate specimens. GeneXpert Dx, GeneXpert Infinity, and/or GeneXpert Xpress systems were used to perform the assays [138].

Table 2 summarizes some commercialized microfluidic platforms for the rapid detection of COVID-19.

The combination of two advanced technologies, namely microfluidics and biosensors, creates a promising diagnostic platform for the timely screening of viral infection. For final commercial applications, manufacturers should consider the design of the device and the compatible integration of each component such as the incorporation of a pump system, power supply, or detection system into one platform for result readout. Importantly, an ideal platform should minimize the interpretation of the operator to reduce errors made by non-expertise. Therefore, the development of a fully integrated product without the need for prior sample preparation is needed. With strong expertise in marketing, the company should provide feedback on consumers’ requirements and the potential of the products in a certain market. In turn, scientists should develop a microfluidic platform that meets all requirements.

## 4. Conclusions

Recently, with the application of biosensors and microfluidic technology, the diagnostic field for infectious diseases caused by viruses has seen great development (Table 3). Routine methods for virus detection such as plate culture, immunoassays, or PCR are usually time-consuming, expensive, and limited to well-developed areas. Conventional methods are not prone to rapid diagnosis and therefore prevent the timely controlling of the spread of viral infection. Microfluidic-based sensors are fast, simple to use, portable, and inexpensive. It is critical that these inventions can reach all corners of the globe, from highly equipped labs to low-resource areas, to screen for viral transmission and control the spread of infection. Although the development of these devices is rapid and promising, there are some limitations in terms of accuracy, sensitivity, and system integration compared to standard detection methods. Real samples, such as clinical samples, contain a complex of components, including some proteins which tend to adsorb nonspecifically to the surface of the microchannels, and the nonspecific adsorption can reduce the accuracy and sensitivity of the assay at low concentrations of analytes. Another challenge in device development is the full integration of all analysis procedures into one single platform. Bulky equipment is usually needed to manipulate and control the fluid inside the microchannel for analysis, hindering the full integration of the microfluidic devices. Future improvements should be implemented to address these problems and to become one of the standard diagnostic tools for the detection of virus infection.

## Figures and Tables

**Table 1:** Commercialized microfluidic products.

Company	Main Products
Roche Diagnostics	Rapid antigens test cobas^®^ SARS-CoV-2 & Influenza A/B test
Cepheid	GeneXpert System Xpert^®^ Xpress SARS-CoV-2
Caliper Life Sciences	LabChip system
Abbott Laboratories	ID Now™ Determine™ HIV-1/2 AG/AB Combo
Standard BioTools Inc.	BioMark HD System X9™ Real-Time PCR System
Agilent Technologies	The AriaDx instrument

**Table 2:** Commercialized microfluidic products for COVID-19 screening.

Platform	Manufacturer	Assay	Target	Analysis Time	Ref.
Elecsys^®^ Anti-SARS-CoV-2	Roche	Immunoassay	SARS-CoV-2 antibodies	18 min	[135]
Cobas^®^ SARS-CoV-2	Roche	RT-PCR	ORF1a/b non-structural region, E gene	20 min	[136]
ID NOW™ COVID-19	Abbott	NEAR	RdRp gene	15 min	[137]
Xpert^®^ Xpress CoV-2 *plus*	Cepheid	RT-PCR	N, E gene	45 min	[138]
1copy COVID-19 qPCR Kit	1drop Inc	RT-PCR	E, RdRp gene	22 min	[139]
Lucira COVID-19 All-In-One Test Kit	Lucira Health	RT-LAMP	N Gene	30 min	[140]
Biosynex COVID-19 Ag+ BSS Rapid Test	BIOSYNEX S.A.	RT-PCR	N-protein	10 min	[141]

**Table 3:** An overview of molecular and immunological diagnostic platforms for virus detection.

Detection Method	Sensing Platform	Virus	Target Analytes	LOD	Assay Time	Year of Publication	Ref.
Immunoassay methods	Immuno-chromatic probe based LFA	Japanese Encephalitis	Non-structural 1 (NS1) secretory protein	10 pg/mL	10 min	2022	[25]
	Magnetic quantum dot nanobeads based LFA	Infuenza A	Virus particle	22 PFU/mL	35 min	2020	[27]
	SER-based LFA	Influenza A	Virus particle	1.9 × 10^4^ PFU/mL	-	2016	[34]
	Portable SERS-LFA system	SARS-CoV-2	Nucleocapsid protein	3.53 PFU/mL	15 min	2022	[37]
	ELISA-based microfluidic chip	SARS-CoV-2	Nucleocapsid (N) protein	0.1 pg/mL	40 min	2022	[51]
	Multicolorimetric ELISA integrated with microfluidic device	Hepatitis C	Core antigen	9.1 ng/µL	50 min	2021	[52]
	Electrochemical immunoassay	SARS-CoV-2	Antigen (Nasopharyngeal samples)	50 PFU/mL	70 min	2022	[57]
	ELISA-based microfluidic immunoassay	SARS-CoV-2	Antigens and antibodies (Serum sample)	0.3 pg/mL	10 min	2021	[59]
	Graphene-based FET biosensing	SARS-CoV-2	Spike protein (Nasopharyngeal samples)	1 fg/mL	-	2020	[65]
Molecular methods	Portable Magnetofluidic real-time RT-PCR instrument	HIV	RNA extraction (Whole blood sample)	500 copies/mL	15 min	2023	[88]
	“FilmArray”: Nested multiplex PCR	21 common viral and bacterial respiratory pathogens	DNA/RNA extraction	Coronavirus HKU1 (1.9 × 10^6^ RNA copies/mL)	60 min	2011	[89]
	Continuous-flow digital PCR device	Hepatitis B	DNA extraction (Serum sample)	10^3^ to 10^5^ IU/mL	-	2020	[90]
	Pocket-size Reverse transcription (RT)-LAMP device	SARS-CoV-2	RNA extraction (Oropharyngeal swab samples)	300 RNA copies/reaction	35 min	2021	[98]
	Paper-based colorimetric LAMP device	SARS-CoV-2	Virus particle	200 genomic copies/μL (Saliva sample)	60 min	2021	[99]
	Wearable RPA microfluidic device	HIV-1	DNA	100 copies/mL	24 min	2019	[103]
	RT-RPA and CRISPR-based paper microdevice	SARS-CoV-2	Nucleoprotein (N) gene and spike (S) gene	10^2^ copies viral RNA/test	60 min	2021	[123]
	Microfluidic Chip-Powered CRISPR/Cas12a System	Multiple respiratory viruses	DNA/RNA extraction (Swab samples)	50–200 copies/mL	<40 min	2022	[125]
	Graphene-based FET biosensing	SARS-CoV-2	Unamplified nucleic acids (Nasopharyngeal samples)	1–2 copies/100 μL	<4 min	2022	[129]

## Data Availability

No new data were created or analyzed in this study. Data sharing is not applicable to this article.
